# The Effect of Nanoparticle-Incorporated Natural-Based Biomaterials towards Cells on Activated Pathways: A Systematic Review

**DOI:** 10.3390/polym14030476

**Published:** 2022-01-25

**Authors:** Nur Izzah Md Fadilah, Isma Liza Mohd Isa, Wan Safwani Wan Kamarul Zaman, Yasuhiko Tabata, Mh Busra Fauzi

**Affiliations:** 1 Centre for Tissue Engineering and Regenerative Medicine, Faculty of Medicine, Universiti Kebangsaan Malaysia, Jalan Yaacob Latiff, Bandar Tun Razak, Kuala Lumpur 56000, Malaysia; nurizzahfadilah@gmail.com; 2 Department of Anatomy, Faculty of Medicine, Universiti Kebangsaan Malaysia, Jalan Yaacob Latiff, Bandar Tun Razak, Kuala Lumpur 56000, Malaysia; ismaliza.mohdisa@ukm.edu.my; 3 Department of Biomedical Engineering, Faculty of Engineering, Universiti Malaya, Kuala Lumpur 50603, Malaysia; wansafwani@um.edu.my; 4 Centre for Innovation in Medical Engineering, Department of Biomedical Engineering, Faculty of Engineering, Universiti Malaya, Kuala Lumpur 50603, Malaysia; 5 Laboratory of Biomaterials, Department of Regeneration Science and Engineering, Institute for Frontier Life and Medical Sciences, Kyoto University, Kyoto 606-8397, Japan; yasuhiko@infront.kyoto-u.ac.jp

**Keywords:** nanoparticles, nanotechnology, natural biomaterials, mechanisms, cells, signaling pathways, regenerative medicine, wound healing

## Abstract

The advancement of natural-based biomaterials in providing a carrier has revealed a wide range of benefits in the biomedical sciences, particularly in wound healing, tissue engineering and regenerative medicine. Incorporating nanoparticles within polymer composites has been reported to enhance scaffolding performance, cellular interactions and their physico-chemical and biological properties in comparison to analogue composites without nanoparticles. This review summarized the current knowledge of nanoparticles incorporated into natural-based biomaterials with effects on their cellular interactions in wound healing. Although the mechanisms of wound healing and the function of specific cells in wound repair have been partially described, many of the underlying signaling pathways remain unknown. We also reviewed the current understanding and new insights into the wingless/integrated (Wnt)/β-catenin pathway and other signaling pathways of transforming growth factor beta (TGF-β), Notch, and Sonic hedgehog during wound healing. The findings demonstrated that most of the studies reported positive outcomes of biomaterial scaffolds incorporated with nanoparticles on cell attachment, viability, proliferation, and migration. Combining therapies consisting of nanoparticles and biomaterials could be promising for future therapies and better outcomes in tissue engineering and regenerative medicine.

## 1. Introduction

**Figure 1:**
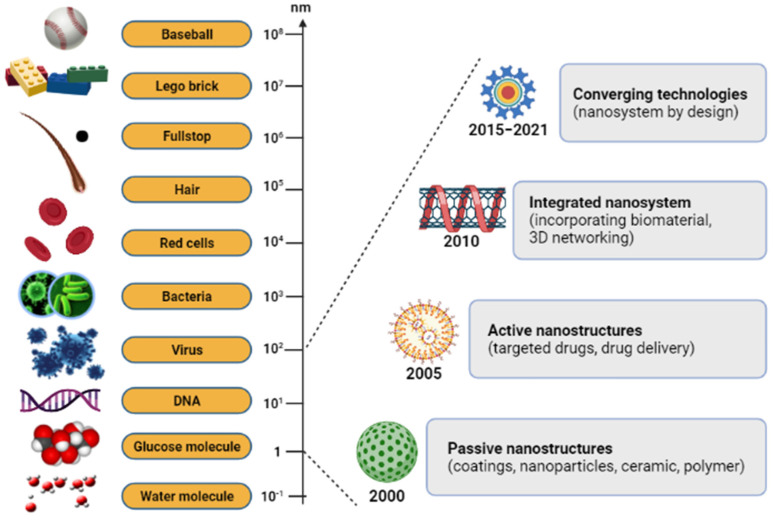
The relative size scale of macro-, micro-, and nanoscopic objects with the timeline for the nanotechnology development.

Nanoparticle-based therapies have a wide range of applications nowadays, and advances in nanotechnology offer novel solutions to disease problems. Recently, nanodelivery systems are rapidly developing new materials in the nanoscale range that are employed to deliver therapeutic agents to specific targeted sites in a controlled manner. It has offered numerous exciting possibilities in healthcare, and a few products are now available on the market. Nanotechnology is essential technology in the 21st century, with an atomic group at the nano-scale size of 1–100 nm [1,2]. We can obtain it from natural sources, chemically synthesize it, or obtain it as one of the by-products of forming nanoparticles [3,4]. The nano-scale science and engineering fields are consolidated under a unified science-based definition and a twenty-year research and development vision for nanotechnology (Figure 1) [5]. There has been a steadily growing interest in using nanoparticles over the last few years. In modern-day medicine, nanoparticles have become an indispensable tool for bioactive agent delivery and can be used in disease monitoring therapy [6]. Generally, nanoparticles were studied because of their size-dependent physical and chemical properties. Besides displaying nanoparticles, there are other examples of the products in nano-scale technology, such as nanofibers and nanopatterned surfaces, which have also been used to direct cell behavior in various biomedical applications [7]. The advantages of nanoparticle characteristics and composition, including a high surface area with adjustable surface properties and high penetration ability, make it one of the widely preferred candidates [8]. Notwithstanding the other possible benefits, a significant advantage of nanoparticles is their beneficial size and shape, with the ability to improve their appearance. An assortment of nanoparticle-organized materials can be developed and applied today, and can benefit researchers’ ability to observe at a cellular level without causing significant interference. Based on the success of nanoparticles in passing through cell membranes, biomaterials with natural properties functioning with other suitable features can be constructed and applied [9].

Biomaterials are classified into two categories, the first of which includes synthetic polymers including polyvinyl alcohol (PVA) [10], polyethylene glycol (PEG) [11], and polylactide (PLA) [12]. Meanwhile, the second category is from natural-based polymers such as cellulose [13], collagen [14,15], gelatin [16], chitosan [17], and silk fibroin [18] which are mainly from animals and plants. Natural-based biomaterials are easily accessible, usually non-toxic and non-immunogenic, besides displaying excellent biocompatibility and biodegradability properties, which have been pinpointed (Table 1) [19]. However, natural-based biomaterials have certain drawbacks due to their weak mechanical strength, without a suitable crosslinker as a supporting component to enhance the stability and assembly of complex structures from the fabricated biomaterials [20]. Moreover, natural-based biomaterials, particularly collagen has been abundantly found in the extracellular matrix (ECM) of human tissue [21]. Consequently, they are intrinsically able to point to cell identification sites that allow them to interact with the surrounding cells and ECM. Because they are chemically compatible and have functional groups, they can incorporate with nanoparticles or conjugate with other target molecules. These chemical modifications will affect their solubility, permeability, charge, circulation time, loading efficiency, and interaction with target receptors [22]. An overall safety assessment, however, is necessary before their clinical application. The use of natural-based biomaterials incorporated with nanoparticles is also an attractive approach that is most desirable for regenerative tools.

Strategies for applying the biomaterials to cure or treat diseases can be achieved in one of two approaches: either with the features of the nano-scaled materials used, or with the materials as a carrier molecule, to deliver active compounds pharmaceutically to the specific site [23]. Up until now, researchers have focused on nanoparticle-based biocompatible materials, which are increasingly prevalent and used in a variety of potential biomedical engineering applications, including drug delivery systems [24], wound healing [25,26], tissue engineering [20,27], dentistry [28,29], cancer therapy [30] and other related research areas. Despite their interesting applications, research into the use of nanoparticles as biomaterials is also broad [31,32,33]. The nanoparticle-incorporated biomaterials provide a perfect composite material, demonstrating new or improved properties and activated therapeutics. The structural properties of these composite biomaterials, and the arrangement of each constituent of nanoparticles, synergistically enhance biomedical capabilities. By discovering nanoparticle-incorporated natural-based biomaterials, this has great implications for approaches involving biological subjects. The ability to modify the properties of biomaterials methodically by monitoring their structures allows them to be used for the treatment, diagnosis and biological system of regenerative dysfunction [34].

**Figure 2:**
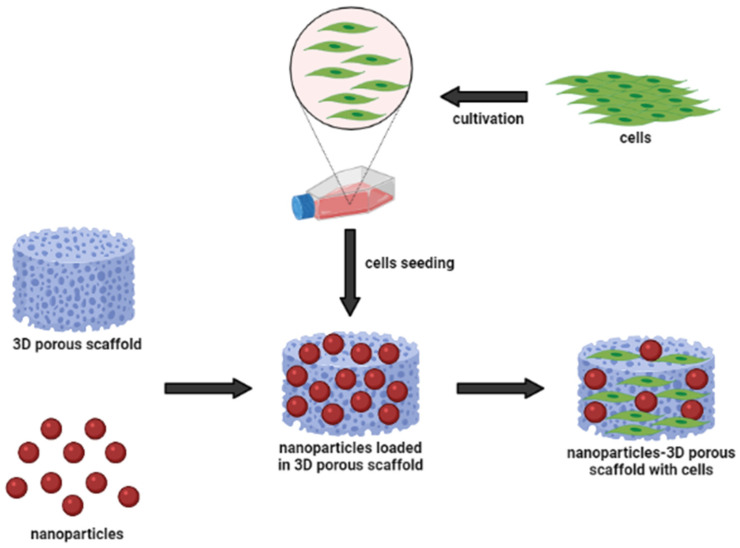
Nanoparticles and biomaterial-based porous scaffold for the delivery of bioactive molecules.

Figure 2 shows a schematic representation of the nanoparticle-incorporated natural-based biomaterial scaffolds for delivering bioactive molecules. In addition, the incorporation of functionalized nanoparticles into a porous sponge, together with cells, has developed a tissue-engineered scaffold for biomedical applications. These nanoparticles can act as nanocarriers to slowly release the cargo (bioactive molecules) to the target site for long-term efficacy. They may influence changes in cell morphology and function, based on the types of biomaterials. Occasionally, nanoparticles can cause inflammatory responses between cell interactions; however, they are easily incorporated into implant design to modulate the inflammatory responses. To develop multi-functional platforms, the incorporation of nanoparticles into biomaterial scaffolds such as hydrogels and electrospun fibers is primarily governed by pore size, which mostly enhance the resulting efficacy of the scaffolds. Hence, we can say that incorporating different types of nanoparticles with advancing biomaterials is of great interest. Investigators and researchers further scrutinize the interactions of nanoparticle-incorporated natural-based biomaterials to detect their cellular response [35,36]. Therefore, it is of utmost importance to understand the underlying mechanisms, including cellular functions and signaling pathways, in mediating wound healing. In the present systematic review, we summarized the results of studies that assessed the biological effects and activated-signaling pathways of nanoparticle-incorporated natural-based biomaterials. Besides, the authenticity and value-added of the present systematic review were to evaluate and compare the current therapeutic efficacy of tissue engineering and regenerative medicine using natural-based scaffolding enhanced with nanoparticle-incorporation. Therefore, it would provide a better understanding for scientist and researchers in determining the importance of nanoparticles in therapeutic delivery, either used directly or embedded in scaffolds.

## 2. Methodology

### 2.1. Literature Search Strategy

A systematic computerized literature search was conducted to determine relevant studies that reported the use of various types of nanoparticles embedded with natural-based biomaterials, and their biological cell effects. The systematic review was compiled based on the Preferred Reporting Items for Systematic Review and Meta-Analyses (PRISMA) guidelines to ensure the quality and transparency of the study [37]. Four databases were searched using Scopus (Elsevier, Amsterdam, The Netherlands), ISI Web of Science (WoS) (Clarivate Analytics, Philadephia, PA, USA), PubMed (National Center for Biotechnology Information-NCBI, Bethesda, MD, USA), and Google Scholar (Mountain View, CA, USA), to find the relevant research articles within the last eight years, from 2014 to 2021. In all databases, we used a combination of controlled terms from MeSH (Medical Subject Headings) and terms guided by the focus question formulated using the PICO strategy, whereby population (P) referred to the laboratory studies on nanoparticles and biomaterials for biomedical engineering applications; intervention (I) referred to nanoparticle-incorporated natural-based biomaterials; comparison (C) referred to types of nanoparticles and biomaterials, and outcome (O) referred to the activation of cellular pathways.

The search strategy or searching method used two sets of keyword combinations, which were: (1) nanoparticle* (to obtain nanoparticle or nanoparticles) or particle* (to obtain particles or particles) or biomaterial* (to obtain biomaterial or biomaterials) or material* (to obtain material or materials) or natural material* (to obtain natural material or natural materials); and (2) cellular pathway* (to obtain cellular pathway or cellular pathways) or signaling pathway* (to obtain signaling pathway or signaling pathways) or biomedical application* (to obtain the biomedical application or biomedical applications) or regenerative medicine application* (to obtain regenerative medicine application or regenerative medicine applications). The comprehensive search strategy is summarized accordingly in Table 2.

### 2.2. Inclusion Criteria

Due to limited translation resources, we only considered published research articles written in English. The research articles that reported the effect of nanoparticle-incorporated natural-based biomaterials, with the main priority of biomedical engineering or regenerative medicine applications, were included in this review, including the different types of nanoparticles and biomaterials. Moreover, the articles that revealed the cellular functions associated with activated signaling pathways in cells, including fibroblasts, keratinocytes and endothelial cells, were also reviewed.

### 2.3. Exclusion Criteria

The exclusion criteria included research articles that were submitted or written in a language other than English. All editorials, conference papers, news, case reports, review papers, and letters were also excluded.

### 2.4. Data Extraction and Management

**Figure 3:**
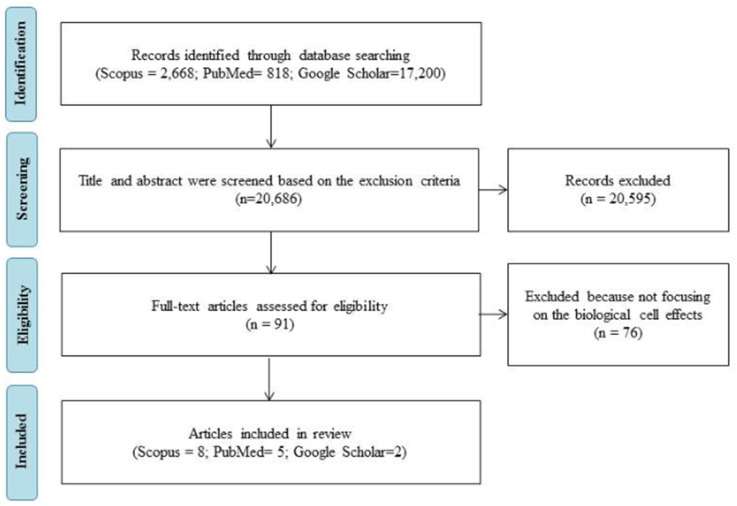
Flow diagram of article selection and data extraction management through the different steps of a systematic review.

All research articles were screened in three steps to meet the requirement of this systematic review. Firstly, a title screening step was applied to remove titles that did not match the inclusion criteria. Secondly, an abstract examination of the remaining papers was conducted, to remove inappropriate articles based on the inclusion criteria. The final step included was removing article papers that did not focus on the biological cell effects (activated pathways). A keyword search returned 91 articles that might potentially be relevant. Once selected, a total of 15 articles were chosen for review after determining if they met the criteria. A flow chart of the article selection process is shown in Figure 3.

### 2.5. Quality Assessment

The review was accompanied by a methodological approach using the critical evaluation for systematic reviews [38]. Both the primary and secondary reviewers discussed each item for each study included in this review. All included studies were considered acceptable for the purpose of this review in terms of specific study characteristics. Previous discussions between independent reviewers were conducted to determine the acceptable level of information for a positive assessment compared to a negative or “unclear” response. A positive response referred to the research articles that had contents which met the requirement for this review.

## 3. Results

### 3.1. Study Characteristics

Among the selected articles, all articles included nanoparticle-incorporated natural-based biomaterials and reported on biological cells in biomedical applications, especially in tissue regeneration and regenerative medicine. All of the studies were published between March 2017 and January 2021. Generally, the selected research articles were identified and discussed for the studies that involved different types of nanoparticles and natural-based biomaterials. We also reviewed the effects of the incorporation of nanoparticles on cellular functions, including cell viability or cytotoxicity, cell proliferation, cell migration, intracellular antibacterial properties, and the main priority in tissue engineering applications involved in wound care management. We also included the effects of the incorporation of nanoparticles in biomaterials on the mediation of cellular signaling pathways towards wound healing, tissue engineering and regenerative medicine. A summary of all of the studies involved is displayed in a data extraction table in Table 3.

### 3.2. Nanoparticles-Incorporated Biomaterial Platforms

The outcomes from the review highlighted the types of biomaterials, fabrication format and advantages of various biomaterials incorporated with nanoparticles for tissue engineering applications. The groups of biomaterials used were cellulose, collagen, and natural polymers including chitosan and alginate. Four formats for the fabrication of nanoparticle-incorporated biomaterials were reported in fifteen research articles, including (1) (n = 6) nanofibers [27,39,41,42,46,52]; (2) (n = 3) nanocomposite scaffolds [40,44,48]; (3) (n = 5) hydrogel [43,47,49,50,51] and (4) (n = 1) films [45].

The use of therapeutic agents has been formulated over time in drug development [53]. However, the direct usage of standard treatment is not without limitations. In the present day, modern health science develops vital sources of biomaterials that are naturally available to treat a wide range of diseases. The application of these biomaterials is considerably increasing in biomedical and clinical fields due to their versatile and multipurpose qualities; for example, they can be used in combination with nanoparticles or drugs to achieve a synergistic effect in treatment [54]. Consequently, the effective fabrication technique of nanoparticle-embedded biomaterials is based upon the nature of the materials used in the preparation process and the expected results of the end product. The functional properties of biomaterials also play an important role in their design, such as pore size, porosity, the level of toxicity, biodegradation, and mechanical strength. A notable example includes the wound dressing material, for which Liu et al. determined the optimal pore size to be in the range of 40–80 µm, and optimal porosity to be ~85%; they determined that this would be suitable for wound healing and regeneration [55].

**Figure 4:**
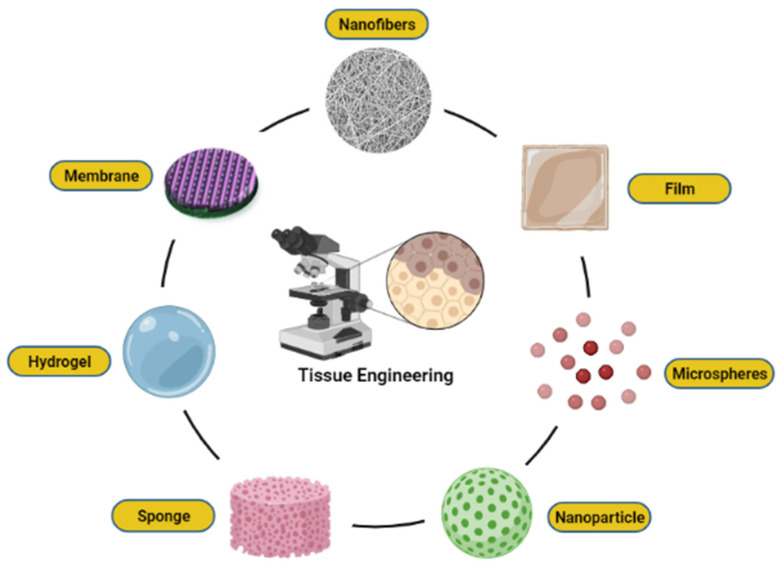
Various formats of biomaterials, including nanofibers, film, microspheres, nanoparticle, sponge, hydrogel, and membrane.

Relying on the nature of the wound healing mechanism, a well-defined pore size of nanoparticles with a highly porous structure can regulate the migration rate of cells and facilitate biological processes, including cellular activity and viability. These features consist of benefits in terms of being able to promote matrix swelling and absorption of wound exudates. Therefore, the generation of endothelial and fibroblasts cells was activated, thus enhancing tissue repair. There are different types of fabrication available, and most of the materials used are naturally derived from polymer and the ECM of tissues which possesses different physicochemical and biological properties. Fabrication technology is widely used to produce scaffold from biomaterials, either synthetic or natural [56]. Various formats of biomaterials such as nanofibers, membrane, hydrogel, film, sponge, nanoparticles, and microspheres can be produced using advanced fabrication and processing techniques, as illustrated in Figure 4. Each of these classes is used for specialized applications in advanced tissue engineering and regenerative medicine. Therefore, depending on the treatment goal, the fabricated biomaterial designs can be altered and possess a high level of biocompatibility, especially in biological systems. With a focus on strategies for nanoparticles embedded natural-based biomaterial design, this subtopic seeks to summarize the recent developments in our understanding of the physical and chemical properties.

#### 3.2.1. Silver Nanoparticle-Embedded Electrospun Nanofibers

With the merits of fungicidal [57,58,59], antiviral [60,61,62,63], and antibacterial [64,65,66] properties, silver nanoparticles (AgNPs) have drawn tremendous academic and industrial interests. Along with the development of nanoparticles, AgNPs have been considered for incorporation into biomaterials due to their strong antibacterial properties [67]. Both have been of great interest recently, due to the dual benefits from AgNPs and the polymer nanofibers themselves. However, their small sizes mean that AgNPs can easily penetrate biofilms and cell membranes; thus DNA will be damaged, and cell proliferation and cellular ATP production will be inhibited [68]. Therefore, AgNPs impregnated into nanofibers would be more efficient than attaching AgNPs directly to the surface treatments.

Electrospinning technology becomes an effective method and reliable technique for producing microfiber or nanofiber [69,70]. Compared with traditional fabrics, polymer nanofiber can offer a significantly higher number of reaction sites, and their permeability could deliver high surface-to-volume ratio and high porosity [71]. These fibers have been widely used in biomedical applications, including in developing wound dressings that reflect their properties, such as large surface area, biomimetic lateral scales, and comprehensive material selectivity [72,73,74]. Specifically, fibers can mimic the three-dimensional (3D) network of the native skin ECM, given that it is a perfect microenvironment for cell adhesion, proliferation, and differentiation [75]. In a previous study, Eghbalifam et al. [76] reported that 3 wt% of AgNPs loaded nanofibrous PVA/PCL mat had cell growth capability. Their research demonstrated that the cell viability of the Ag sample on days 5 and 7 was not significantly different from that of the reference cells. They concluded that the introduction of AgNPs did not affect the surface cellular biocompatibility of fibers.

These findings were similar to those summarized by El-Aassar et al. [77], where the polygalacturonic acid (PGA)/hyaluronic acid (HA)/poly(vinyl)alcohol (PVA) nanofiber embedded with AgNPs became very effective against Gram-positive bacterial strains as well as a Gram-negative bacterial strain. However, the study did not conduct in vitro cell evaluation and the statistical significance of the percentage of cell viability was not described. Interestingly, nanofiber mats affixed with AgNPs were applied to the injury site of albino rats in vivo. The positive results demonstrated significant wound healing from day 8 onwards and an epithelialization period on day 14. It unraveled maximum wound epithelialization and collagen deposition after 14 days of nanofiber administration.

#### 3.2.2. Chitosan-Based Nanocomposite Porous Bioscaffolds

New nanoparticle- or nanomaterial-based treatments for skin lesions have been developed over the years. The nanocomposites are generally used as 3D nano-fibril structures called scaffolds. These can be divided into porous materials, colloids, copolymers, and gels [78]. Plenty of studies have established the use of nanoparticles as effective molecules of nanocomposite scaffolds in tissue engineering applications [79,80,81]. Biodegradable polymers can have a plethora of different functions, potentially making them an ideal scaffolding material. An example that can be mentioned of a composite material is chitosan, a natural polysaccharide that has the ability for gel formation, making it useful in many biomedical applications. In particular, it is known to be beneficial for wound healing by stimulating tissue regeneration, increasing blood coagulation, facilitating the rate of O_2_ transmission, preventing microorganisms, and accelerating epithelialization [82,83]. It has fascinating antibacterial activity towards gram-positive and gram-negative bacteria, but its solubility severely limits the antibacterial activity. Therefore, the chemical modification of chitosan, by combining it with other antibacterial compounds, will also improve the properties of bacteria inhibition [84]. Similarly to this feature, with weak mechanical strength, chitosan has to be impregnated with nanoparticles to build up its structure [85]. It was later found that the reinforcement of nanocrystal particles with chitosan could improve the mechanical behavior and dimensional stability of the nanocomposite. To the best of their knowledge, Mariia et al. [86] reported that chitosan-incorporated cellulose nanocrystals have become a novel nanocomposite that enhance morphological structure and have a strong cytocompatibility. This interaction may be attributed to the maximum thermal stability, up to 128 °C, with a content of 20% cellulose nanocrystals. In addition, a similar conclusion was reported by [87].

#### 3.2.3. Metallic Nanoparticle-Entrapped Hydrogel

The combination of metal nanoparticles in hydrogels is a newly emerging area of research in wound healing, tissue engineering, and regenerative medicine [88]. A hydrogel can be defined as a 3D network of natural or synthetic hydrophilic polymers that are cross-linked together, physically or chemically. They have unique properties, including softness, flexibility, biocompatibility, and high water content [89]. With its properties of high water content, soft consistency and porosity, the hydrogel is able to stimulate naturally and resemble living tissue [90,91]. This scaffolding easily mimics the ECM, acts as structural support for cells, and provides a potential space for new tissue formation, including improved cell attachment, proliferation and differentiation [92]. However, hydrogel has some limitations as a delivery vehicle in promoting tissue growth, including a lack of bioactive properties and weak mechanical strength. To boost up physical, chemical, and biological properties, metal particles—particularly in gold (Au), silver (Ag), and inorganic or ceramic nanoparticles such as silica–hydroxyapatite (HA)—can be incorporated into the hydrogel matrix when forming the nanoparticles-hydrogel scaffolds [93].

**Figure 5:**
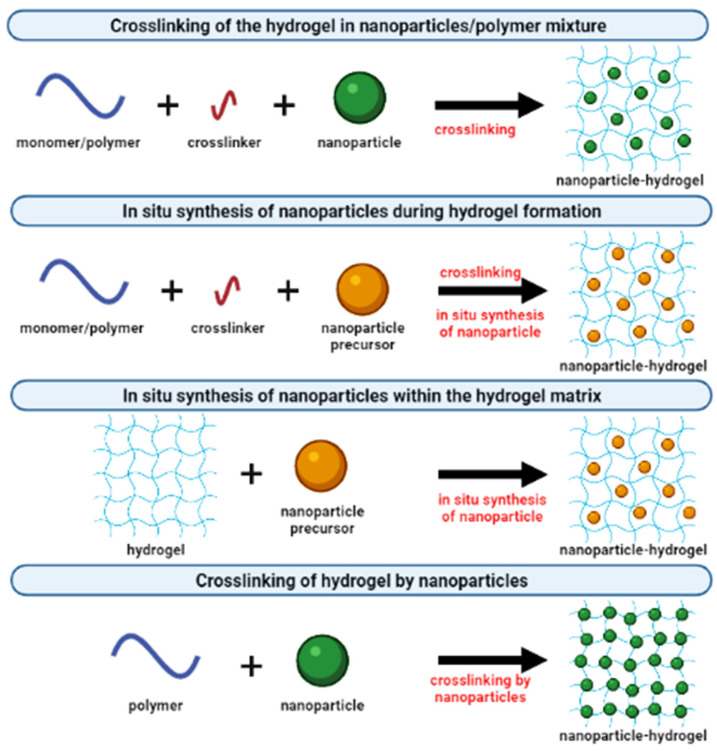
Different approaches to incorporating nanoparticles into cross-linked hydrogel matrices.

Presently, the concept of forming an inorganic–organic framework with improved properties has led to innovation in tissue engineering scaffolds. There are ways to fabricate metallic nanoparticles incorporated into crosslinked hydrogel composites, as summarized in Figure 5. One example of the approaches is the combination of two types of fillers: clay and SiO_2_ nanoparticles, loaded in PVA to produce highly stretchable composite hydrogels. The authors confirm that there are synergistic effects in terms of extensibility due to the integration of both nanoparticles in the polymer network [94]. In addition, another study has been reported on different particle types and their concentrations; the study was about ZnO nanoparticles loaded into an alginate hydrogel. With a lower concentration of ZnO nanoparticles, higher cell viability induced cellular proliferation. A percentage of 1% ZnO demonstrated 88.4% of live cells; however, the nanoparticle dispersion influenced cell viability. From the study, it is unknown whether there is a correlation between toxicity and the concentration of nanoparticles. Nevertheless, cell viability was observed upon incorporation of nanoparticles into the hydrogel, implying the ZnO nanoparticles’ toxicity remains under the threshold for substantial cell damage [95].

Moreover, for the study conducted by Zulkifli et al. [96], the antimicrobial hydroxyethyl cellulose (HEC)-embedded AgNP scaffold exhibited low toxicity, good biocompatibility, and promoted the growth and proliferation of human fibroblast cells. It was essential to show that the scaffolds roughness, provided by AgNPs, supports cell attachment and proliferation over the scaffold. On the other hand, Mahmoud et al. [97] have synthesized gold nanoparticles (AuNPs) with different shapes, loaded into polyethylene glycol (PEG) polymeric hydrogels. The strong antibacterial properties against *S. aureus* and *P. aeruginosa* were also identified. Fascinatingly, the in vivo results showed that the PEG-AuNPs’ hydrogel efficiently healed the wound and completed the wound closure without scars. According to the positive effect of impregnated polymeric hydrogels with nanoparticles on the proliferation of human fibroblasts, they were able to find potential in skin tissue regeneration.

#### 3.2.4. Nanoparticle-Impregnated Polymeric Composite Films

Combined with available treatment strategies, films with a homogeneous polymeric structure were also beneficial in biomedical fields. Recently, it has been suggested that incorporating therapeutic drugs or nanoparticles into the films can enhance the drug’s therapeutic effect and its effectiveness without altering the function of the film, which would be a good idea for skin tissue engineering [98]. Therefore, it is remarkable to explore the performance and biological effects of the film scaffolds incorporated with nanoparticles for wound healing and regenerative medicine applications. A study was conducted on incorporating curcumin into PVA composite films as a viable approach to fabricating a skin-dressing product for wound healing. The combination of collagen on the curcumin/PVA scaffold showed enhanced film formation and increased viability in human skin fibroblast cells. These findings were further supported by the results of their in vivo studies, due to the slow-released effect of impregnated nanoparticles to provide long-term efficacy throughout the progress of wound healing [99]. In another study, a chitosan/gold nanoparticle (AuNPs) film was prepared by blending gold colloid with chitosan solutions. Similarly, the presence of nanoparticles in a polymeric composite film significantly improved the antibacterial properties against *S. aureus* and *E. coli*. The film was also allowed enrichment in cell adhesion and proliferation, due to the specific concentration of nanoparticles [100]. Similarly, the AuNPs promote the expression of cell-related growth factors such as vascular endothelial growth factor (VEGF) and collagen type-1 (COL-1), thus inducing the ECM synthesis; this, in turn, provides a more conducive environment for the cells [101,102]. Moreover, in a study reported by Verma et al. [103], the carboxymethyl cellulose (CMC)/PVA composite film has a good surface roughness for cell adhesion. Meanwhile, the addition of magnesium oxide (MgO) nanoparticles into CMC/PVA polymer film improved antimicrobial property, and the fibroblast cell supportive properties were attained. The highest cell viability was achieved with CMC/PVA polymeric film containing 1.5 wt% MgO nanoparticles. Shortly after these findings, the strategy of incorporating nanoparticles into reservoir-carrying polymer film was shown to have unique advantages, promising outcomes and the functionality of the designed polymer-nanoparticle films.

## 4. Biological Cellular Effects and Signaling Pathways

From the literature search, the findings of the studies were discussed on the effect of the incorporation of nanoparticles into biomaterial scaffolds towards biological cellular pathways. We focused on the observation of its application in wound healing, tissue engineering, and regenerative medicine. A variety of natural polymers have been successfully engineered to associate with different kinds of nanoparticles, resulting in improved efficiency. For this reason, a superior understanding is required of the therapeutic effects of the biomaterial system with intercellular, transcellular, and other activated signaling pathways.

The study of the effects on cell toxicity of nanoparticles embedded in the biomaterials is of great significance. There has been increasing evidence that the biological properties of the scaffold affect various types of cellular behavior, including cell compatibility, viability, proliferation, and migration [104]. To address another important pathway of nanoparticle-incorporated biomaterials towards skin penetration, Kumar et al. [105] discussed the outcomes of using natural compound particles with incorporated biomaterials in skin tissue engineering applications. Based on available knowledge from the findings, the researchers used nanoparticles as carriers, due to their biological, electrical, mechanical and antibacterial properties [36]. As nanoparticles are implanted in the scaffold, it was found to develop the cytocompatibility of the particle substitute material and provide an appropriate surface morphology to enhance cellular functions. The biocompatibility and porous structure of scaffolds allow the cells to adhere to the surface of the scaffold effectively. Biological assays, such as lactate dehydrogenase (LDH) and MTT 3-(4,5-dimethylthiazol-2-yl)-2,5-diphenyltetrazolium bromide (MTT) assays, showed the proper cell attachment and proliferation of the prepared scaffolds. Both MTT and LDH assays are indirect tests known as the primary method for determining the toxicity of compounds with the scaffold, and number of damaged cells released into the culture media, respectively [26].

Due to special distributions of incorporated nanoparticles within the scaffolds in skin tissues, scientists also studied the effect of nanoparticles on cell proliferation and migration to skin cells. In the application of wound healing, recent studies based on the in vitro scratch experiments and in vivo animal studies have demonstrated positive results. The incorporation of nanoparticles into natural-based polymeric biomaterials significantly promoted cell proliferation and migration, which are the biological functions crucial for wound site closure, as well as restoration of barrier function [106,107,108]. The authors shared their findings, indicating that the cell migration path in the scratch area was shortened, and the migration rate increased after using their scaffoldings, which demonstrated excellent performance. These have shown that nanoparticles can act as a biological molecule; meanwhile natural-based biomaterials can be used as a substrate for cell growth, stimulating proliferation and migration. Accordingly, nanoparticles themselves promoted the proliferation and migration of human epidermal cells and human fibroblasts in vitro. Their proliferative-promoting effect always depends on their concentration and duration of action [109]. In addition, the combined application of nanoparticles and biomaterials had a synergistic promoting effect on human epidermal cell proliferation and wound healing. This is possible due to the scaffolds, which can absorb wound exudate and maintain a moist environment, necessary for cellular proliferation at the wound site. After these discoveries, the hydrophilic nature of the natural-based biomaterials’ polymer matrix characteristics was shown to be close to the value of fibroblast proliferation and migration [110].

**Figure 6:**
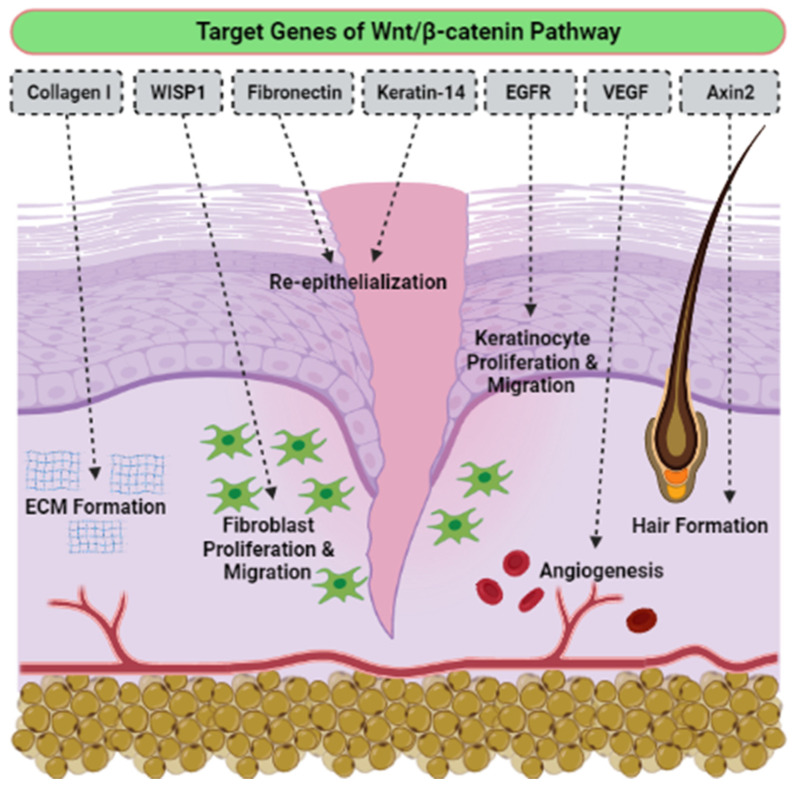
The effects of Wnt/β-catenin activated pathway, targeting genes in wound healing.

Even though there is no determination of signaling pathways in the selected articles, we have accumulated evidence of the activation signaling pathways that are critically involved in proliferation, migration cells, and wound healing. To the best of our knowledge, the activation of the Wnt/β-catenin signaling pathway plays a vital role in the proliferative phase of wound healing and tissue regeneration after injury. The Wnt/β-catenin signaling pathway is activated in the dermis of the wound bed immediately after skin injury. β-catenin is a subunit of the cadherin protein complex, where Wnt plays a specific role in the regulation of β-catenin function [111,112,113]. The signaling of Wnt is mediated by multi-protein complexes together, which consist of glycogen synthase kinase-3 (GSK3β), Axin, adenomatous polyposis coli (APC), and casein kinase 1 (CK1) in the cytoplasm. Several target genes have been identified in cutaneous wound healing, as listed in Table 4. The Wnt/β-catenin activation pathway transcriptionally induces target genes such as Collagen I, Axin2, epidermal growth factor receptor (EGFR), Fibronectin, Keratin-14, VEGF and WISP1 during the wound repair process (Figure 6). Essentially, different Wnt/β-catenin signaling target genes contribute to the various events that occur during wound healing. For example, EGFR regulates the proliferation and migration of keratinocytes; meanwhile, collagen-1 plays a role in ECM formation. The up-regulation or rearrangement of Wnt/β-catenin signaling enhances the proliferation and migration of dermal fibroblasts, thus making them into distinct myofibroblasts. Therefore, it helps to reduce the surface area of the growing scar. Furthermore, the activation of the Wnt/β-catenin pathway not only significantly facilitates migration and differentiation of keratinocytes in the epidermal layer, but it also induces angiogenesis and epithelial remodeling, which directly enhances skin wound healing [114,115].

On the other hand, the TGF-β pathway is also involved in skin wound healing and dermal fibrosis. It is elaborated differently in the regulation of healing rate, which depends on the isoforms [116]. For example, TGF-β1 acts as a fibrosis stimulant factor; meanwhile, TGF-β3 regulates anti-scar activity. In adulthood and embryonic development, the Notch pathway controls epidermal cell differentiation [117], maintains skin homeostasis, and promotes angiogenesis [118,119,120]. A previous study reported that the activation of the Sonic hedgehog (Shh) pathway is required, and sufficient for hair follicle neogenesis (HFN) [121]. Nevertheless, the need for Shh regulation may differ in the development of embryonic hair follicles (HF) and adult HFN. If epithelial Wnts are sufficient for embryonic HF development, both epithelial and dermal Wnts are still required for adult HFN [122,123]. Thus, targeting activated signaling pathways could provide an effective therapeutic approach for regenerative skin wound healing.

## 5. Discussion

The findings from this evidence-based review reported a trend of using nanoparticles incorporated with natural-based biomaterials for tissue engineering and regenerative medicines. However, there are still limits on the study of the signaling pathways. Therefore, we discussed the essential characteristics and functional application of nanoparticle-incorporated natural-based biomaterials, the biology of cellular functions associated with multiple signaling pathways in mediating wound healing, and tissue engineering applications as well. Polymer natural-based biomaterials show high benefits that are well-documented in the literature [124,125]. A large number of the studies showed positive outcomes from physical and biological aspects (cellular interactions), for nanoparticles incorporated with scaffolding compared to scaffolding without nanoparticles incorporated [126,127]. From our observations, we believe that natural-based biomaterials containing nanoparticles function well as scaffolding, and become very important for repairing or regenerating diseased cells and tissues. Indeed, the differences in the results may be due to the variation in parameters such as the size of the nanoparticles, and the scaffold’s roughness.

However, this combination therapy of nanoparticle-biomaterial scaffolds is clinically limited, especially in overcoming the ongoing challenge of the uncontrolled release of nanoparticles from the scaffold; it is also of concern. The nanoparticles themselves may not just be directly taken up by the cells in exposed organs, but may also translocate to the other organs, resulting in undesirable toxicity [128]. With the expanding knowledge of cellular interaction and its mechanisms of action, different cell types demonstrate different cytotoxic responses. For example, a hydrogel containing antimicrobial AgNPs was found to exhibit a different level of toxicity (lower) against human fibroblast to the keratinocyte cell line and their primary cells. This finding indicated that we could achieve more accurate biocompatibility test results using the appropriate cell types [129]. Therefore, a scaffold’s safety assessment should be performed extensively on the affected tissues or organs, and not be limited only to the site of tissue regeneration. 

It is important to determine the molecular and cellular mechanism, specifically in normal skin repair. Early stages of wound healing include hemostasis and activation of keratinocytes and inflammatory cells. The intermediate stage involves proliferation and migration of keratinocytes, proliferation of fibroblasts, matrix deposition, and angiogenesis. Late-stage healing involves remodeling of the ECM, resulting in scar formation and restoration of the barrier. This spatiotemporal process is tightly controlled by multiple cell types that secrete numerous growth factors, cytokines, and chemokines to achieve closure and functional restoration of the barrier [130]. In addition, the signaling pathway of Wnt is reported to have a role in metabolic homeostasis and in cell proliferation. The incorporation of Lanthanum phosphate nanoparticles into the chitosan scaffold showed increasing expression of β-catenin, and this enhancement suggested up-regulation of the Wnt/β-catenin signaling pathway. This would stimulate and improve the cell proliferation [131]. Basically, repairing of skin tissue is regulated at cellular and molecular levels. Although pharmaceuticals that target the Notch and Hedgehog signaling pathways have been tested in clinical trials, pharmaceuticals regulating Wnts are currently not yet tested in a clinical setting [132]. For that reason, a comprehensive approach for the preclinical phase of drug discovery is required in the future.

Nevertheless, none of the articles presented human clinical trial data, implying the efficacy of the nanoparticle-incorporated biomaterials. Since the development of biodegradable nanotechnologies has also increased in the use of naturally based biomaterials towards clinical applications, it encourages the interaction between cells and biomaterials. We recommend that more researchers investigate and address in vivo studies and clinical trials of the functional applications of nanoparticle-loaded natural biomaterials in tissue engineering and regenerative medicine.

## 6. Conclusions and Future Perspectives

In conclusion, this evidence-based systematic review generally shows positive outcomes towards nanoparticles, which are useful for therapeutic delivery of biomedical applications, including in wound healing. All of the above-reported results strongly indicate that natural-based products that incorporate nanoparticles are effective and safe for use in treatment. However, there are still some health hazard concerns, especially regarding toxicity due to uncontrollable use and release. With this in mind, the incorporation of nanoparticles with natural-based biomaterials should be considered, to make the use of nanoparticles’ slow-release, easier, safer and more environmentally friendly. This study also identifies mechanism through which nanoparticle-incorporated biomaterials affect cellular and molecular signaling pathways. Our findings suggest that Wnt/β-catenin signaling pathways play important roles in tissue regeneration, specifically in cutaneous repair. The interaction between the two pathways might play a vital role in the regulation of healing. By understanding the mechanism involved in the signaling pathways for wound healing, we can identify new targets for the development of regenerative wound healing using nanoparticles incorporated into natural-derived biomaterials, combined with the therapeutic agents. For a future perspective, we can design new technologies to mimic native tissue with 3D networking as effective wound healing therapies, if we better understand these complex interactions. This also includes the cost effectiveness and sustainability of the technology with the use of intelligent biomaterials that can revolutionize the field of tissue engineering and regenerative medicine in the future.

To date, bioscaffold-based strategies developed for therapeutic applications present many challenges, and also new opportunities. Recent advancements in natural-based biomaterial synthesis have enabled researchers and scientists to incorporate many nanoparticles, thus facilitating better tissue regeneration with fewer toxicity effects. Despite these recent improvements, more efforts should be made to advance the fabrication of bioscaffolds through a combination of nanoparticles and biomaterials. Tailor-made therapies will become increasingly relevant in coming days. In the future, more clinical trials and in vivo studies will help us to realize the widespread commercial use of natural-based scaffolds incorporating nanoparticles with cost-effectiveness and ease of use.

## Figures and Tables

**Table 1:** Summary of advantages and disadvantages of natural-based biomaterials.

Advantages	Disadvantages
Biodegradability (natural degradation mostly can occur in the body)	Low mechanical strength
High biocompatibility	Possible immune reaction
Interaction with other cells/molecules	
Ability to support cell adhesion, migration, proliferation and differentiation	
Low toxicity	
Lower costs	

**Table 2:** Search strategy for systematic literature review of all databases. (*: to obtain both singular and plural forms of the search criterion).

No	Keywords
1	Nanoparticle *
2	Particle *
3	Biomaterial *
4	Material *
5	Natural material *
6	Cellular pathway *
7	Signaling pathway *
8	Biomedical application *
9	Regenerative medicine application *
10	Or/1–5
11	Or/6–9
12	And/10 & 11
13	Limit 12 to period: 2014–2021

**Table 3:** A summary of studies of natural-based biomaterials embedded with nanoparticles for biomedical applications.

Author/Year	Types of Biomaterials	Nanoparticle Used	Fabrication Format	Study Design	Application	Study Measure Outcome/Biological Effects	Conclusions
Sofi et al., 2021 [39]	Cellulose	Hydroxyapatite (HAp); 0.5–1.5 wt% Silver nanoparticles (Ag NPs); 3.0–7.0 wt%	Nanofibers	In vitro	Tissue engineering	Cell viability Antimicrobial activity	Nanofiber mat (1.5% HAp and 7% Ag NPs) was toxic to growth and proliferation of the fibroblast.
Kaparekar et al., 2020 [40]	Collagen-fibrin	Gallic acid (GA) Chitosan (CSNPs); 0.1–0.5 wt%)	Nanocomposite scaffolds	In vitro In vivo	Wound healing	Cell viability Cell toxicity Cell migration	There was increased collagen deposition, angiogenesis, epithelialization, and fibroblast migration in the GA–CSNPs scaffold treated group.
Ibrahim et al., 2020 [41]	Carboxymethyl chitosan (CMCS) Polyvinyl alcohol (PVA)	Gold nanoparticles (AuNPs); 0.35–1.09 wt%	Nanofibers	In vitro	Medical biomaterials	Antibacterial activity Cell viability	AuNPs capped by CMCS showed lower cytotoxicity, and its antibacterial activities were increased by increasing AuNPs wt% in the nanofibers.
Augustine et al., 2019 [42]	Polycaprolactone (PCL)	Yttrium oxide (Y_2_O_3_)	Fibers	In vitro In vivo	Tissue engineering	Behavior of cells Cell viability Cell proliferation and migration Angiogenesis Inflammatory response	Y_2_O_3_ nanoparticles can perform a vital role in tissue engineering scaffolds to promote cell proliferation and angiogenesis.
Barros et al., 2019 [43]	Alginate	Nano hydroxyapatite (nanoHA); 30–70 wt%	Hydrogel	In vitro Ex vivo	Bone regeneration	Metabolic activity Cell proliferation Cell morphology	The biological response of composites was influenced by nanoHA content: NanoHA 30 wt% enhanced cells proliferation; NanoHA 50 wt% and 70 wt% impaired biological response.
Shams et al., 2018 [44]	Poly-L-lactic acid (PLLA)	Bioactive glass nanoparticles (BGn)	Nanocomposites	In vitro	Medical biomaterials	Cell attachment Cell viability	PLLA nanofibers with BG nanoparticles caused improved cell behavior, including cell attachment, growth, and proliferation.
Liu et al., 2017 [45]	Chitosan/gelatin	Zinc ions (Zn); 5–40 wt%	Multilayer films (layer-by-layer; LBL)	In vitro	Medical biomaterials	Cell viability Cell morphology Bacterial growth	The optimal modified Ti substrate (Ti-LBL-Zn10) had the greatest potential for promoting osteoblast growth.
Nekounam et al., 2021 [27]	Polyacrylonitrile (PAN)	Silica nanoparticles (SNPs); 1–10 wt%	Nanofibers	In vitro	Tissue engineering	Cell cytotoxicity Cell proliferation	The cytotoxicity and proliferation assays showed a noticeable enhancement in the biological features of the NFs/SNPs composite.
Fahimirad et al., (2021) [46]	Polycaprolactone (PCL)	Curcumin (CUR) encapsulated Chitosan (CS)	Nanofibers	In vitro In vivo	Wound healing	Antibacterial activity Cell viability/proliferation Wound healing abilities	Potential application of PCL/CS/CUR with CURCSNPs as an effective novel wound dressing with significant antibacterial activity.
Liu et al., 2020 [47]	Catechol-chitosan (CA-CS)	Zeolitic imidazolate framework-8 nanoparticle (ZIF-8 NP); low (L), medium (M), high (H)	Hydrogel	In vitro In vivo	Bone regeneration	Cell proliferation Bacterial adhesion Osteogenic stability	Among the CA-CS/Z hydrogels, the CA-CS/ZM hydrogel showed acceptable adhesion properties and antibacterial properties, enhancing the stability of the implanting environment after bone transplantation and promoting the healing process of bone defects.
Konop et al., 2019 [48]	Keratin (fur keratin-derived powder; FKDP)	Silver nanoparticles (AgNPs)	Nanocomposite scaffolds	In vitro In vivo	Wound healing	Cell viability Cell migration	FKDP–AgNPs dressing consisting of an insoluble fraction of keratin, which is biocompatible, significantly accelerated wound healing in a diabetic mouse model.
Zhang et al., 2019 [49]	Polyethylene glycol diacrylate (PEG/DA)	Polydopamine/Puerarin nanoparticles (PDA/PUE)	Hydrogel	In vitro In vivo	Wound healing	Cell viability Intracellular antioxidation	PEG-DA/PDA/PUE hydrogels were conducive to cell growth and could accelerate wound healing.
Masood et al., 2019 [50]	Chitosan–Polyethylene glycol (CH-PEG)	Silver nanoparticles (AgNPs)	Hydrogel	In vitro In vivo	Wound healing	Antibacterial property Antioxidant property Re-epithelialization	Silver nanoparticle impregnated chitosan–PEG hydrogel can be a promising material for wound healing dressing for chronic diabetic wounds.
Kalantari et al., (2020) [51]	Polyvinyl alcohol—Chitosan (PVA/CH)	Cerium oxide nanoparticles (CeO_2_-NPs); 0–1 wt%	Hydrogel	In vitro	Wound healing	Cell viability Cell metabolic activity Antibacterial activity	The chitosan/PVA hydrogels incorporated with CeO_2_-NPs could be a potential candidate as a robust wound dressing agent that, impressively, may decrease wound infections without resorting to the use of antibiotics.
Norouzi et al., 2021 [52]	Polyvinyl alcohol (PVA)	Zinc oxide (ZnO)	Nanofiber	In vitro In vivo	Wound healing	Cell viability Antibacterial activity Keratinocyte migration	ZnO nanoparticles were responsible for accelerated epithelial regeneration and better cell attachment. Therefore, these composite fibers have potential in biomedical applications such as wound healing and tissue reconstruction.

**Table 4:** Target genes involved in the Wnt/β-catenin signaling pathway, which is related to wound healing.

Wnt Target Genes	Role in Wound Healing
Axin2	Hair formation through activation of hair follicles
Collagen I	Key protein of ECM synthesized during the proliferative phase
Collagen III	Key protein of ECM synthesized during the early proliferative phase
EGFR	Regulation of keratinocyte migration to wound bed
Endothelin-1	Regulation of fibrosis and calcification
Fibronectin	ECM formation and re-epithelialization
Keratin-14	Re-epithelialization
VEGF	Stimulation of angiogenesis
WISP1	Promotion of dermal fibroblast proliferation and migration

## Data Availability

The data presented in this study are available on request from the corresponding author.
